# Effect of N6-methyladenosine methylation-related gene signature for predicting the prognosis of hepatocellular carcinoma patients

**DOI:** 10.3389/fsurg.2023.1052100

**Published:** 2023-03-03

**Authors:** Xinyu Zheng, Yingyue Zhang, Yun Wang, Zijing He, Qiang Zhang, Dapeng Ren, Xiao Yan, Xiao Yuan

**Affiliations:** ^1^ Department of Orthodontics, The Affiliated Hospital of Qingdao University, Qingdao, China; ^2^ School of Stomatology of Qingdao University, Qingdao, China; ^3^ QiLu Hospital of Shandong University, Qingdao, China

**Keywords:** hepatocellular carcinoma, prognostic model, m 6 a methylation-related genes, TCGA database, immunohistochemical staining experiments, biomarker

## Abstract

**Background and aims:**

Hepatocellular carcinoma (HCC) is a common cause of cancer-related death in humans. Increasing evidence indicates that an imbalance in N6-methyladenosine (m6A) methylation is linked to the occurrence and development of cancer. We then developed a prognostic model as an independent risk factor with which predict the prognosis of HCC.

**Methods:**

We obtained the gene expression and clinical data of HCC patients from the TCGA databases. The prognostic value of m6A methylation-related genes in patients who had HCC were subjected to comprehensive bioinformatics analysis. We use $\mathrm{Risk}\;\mathrm{Score}=\sum_{i=1}^{n}\mathrm{Coe}f_{i}\times X_{i}$ to construct the risk scoring formula. We collected pathological specimens from 68 patients who had HCC, and conducted immunohistochemical staining experiments on the specimens.

**Results:**

There was a significant correlation between candidate m6A methylation-related genes (YTHDF2, METTL14 and ZC3H13) overall survival of HCC patients. Among the 68 HCC patient specimens that underwent immunohistochemical staining, all cancer tissues were positive for METTL14, YTHDF2, and ZC3H13 staining in contrast to the adjacent tissues. We conducted a Kaplan-Meier survival analysis. The results showed that patients who had low METTL14 expression had a longer survival time than those of patients who had high METTL14 expression. Also, patients with low YTHDF2 expression had a longer survival time than patients with high YTHDF2 expression. Finally, patients with high ZC3H13 expression lived longer than those with low ZC3H13 expression. This result is consistent with the bioinformatics analysis conclusion above.

**Conclusions:**

Generally, the prognostic model that was based on m6A methylation-related genes in this study can effectively predict the prognosis of HCC patients.

## Introduction

HCC, one of the most common cancers in Asia, is a common cause of cancer-related death in humans (1). It is estimated that in 2018, there were approximately 42,220 new cases of cancer in the liver and intrahepatic bile ducts and 30,200 deaths in America (1). Moreover, the overall survival(OS) rates of patients with HCC are only 36% and 17% at one year and three years, respectively (1). Although great progress has been made in surgical resection, radiofrequency ablation, systemic therapy, and liver transplantation for treating HCC, the prognoses of HCC patients continues to be poor (2, 3). In addition, HCC patients with who have the same tumor stage, or other similarities in clinicopathological characteristics, sometimes may have different prognoses due to individual differences (1, 2). Therefore, it is vital to develop new biomarkers to predict precisely the prognosis of HCC patients.

N6-methyladenosine (m6A) methylation is the most common RNA modification of human cells (4). Extensive research has shown that m6A methylation regulates cellular processes. This includes cell self-renewal, differentiation, invasion and apoptosis. It accomplishes thus by modulating gene expression (5). Modulators of m6A methylation play a significant and pleiotropic role in the regulation of HCC. Both mRNA and ncRNA are involved in m6A-mediated biological processes in HCC (6). Some studies have found that the stability and degradation of mRNA are the most common m6A-driven regulatory functions in HCC (6, 7). In addition, studies have shown that m6A modification regulates ncRNAs in various ways, including splicing, preprocessing, stability and decay. Thus, ncRNAs affect and participate in the m6A process of HCC (7). According to the literature, regulators of m6A modification include “writers.” The writers are methyltransferases that are mainly responsible for transferring the methyl group to the N6 position; “readers.” The readers are RNA-binding proteins that regulate RNA functions by recognizing specific m6A-modified positions; and “erasers.” The Erasers are demethylases that remove the methyl group. m6A methylation-related genes, including “writer” genes (RBM15/15B, METTL3, METTL14, WTAP VIRMA and ZC3H13), “reader” genes (IGF2BP1/2/3, YTHDC1/2, YTHDF1/2/3, HNRNP and eIF3), and “eraser” genes (ALKBH3, ALKBH5 and FTO). The eraser genes maintain cooperatively maintain the dynamic and reversible balance of m6A methylation (8, 9).

Upon consulting the literature, we found some applications of m6A methylation in HCC (10). METTL3 (methyltransferase-like 3) is related to the poor prognosis of HCC patients (10, 11). It promotes the proliferation, migration and colony formation of HCC cells by posttranscriptional silencing of SOCS2, which depends on YTHDF2 (YTH domain containing 2) (12–14). In addition, METTL14 (methyltransferase-like 14) is a reader that has a beneficial role in HCC by regulating m6A-dependent miRNA processing (10). MiR-145 downregulates YTHDF2 by targeting the 3′UTR (15). In summary, the upregulation of METTL3 or downregulation of METL14 can predict a poor prognosis of HCC patients and can lead to HCC progression and metastasis (16). METTL3 inhibits the expression of SOCS2 in HCC *via* the miR-145/m6A/YTHDF2 axis (17). These studies provide a new dimension for the investigation of epigenetic changes in liver cancer. In this work, we conducted extensive of transcript and clinical that we obtained from the TCGA and ICGC databases (18). We applied consensus clustering analysis, least absolute shrinkage and selection operator (LASSO) regression analysis, and Cox regression analysis to develop m6A methylation-related gene signatures. We then developed a prognostic model based on m6A modifications as an independent risk factor with which predict the prognosis of HCC and to suggest therapeutic targets for HCC.

## Methods

### Data download and processing

The potential m^6^A methylation-related genes were obtained. These included ZC3H13, METTL14, YTHDC1, YTHDC2, FTO, ALKBH5, KIAA1429, RBM15, METTL3, WTAP, YTHDF1 and YTHDF2 were obtained. RNA-seq transcriptome and clinical data of HCC were downloaded from the TCGA database. Patients were excluded if their survival times, survival status and clinicopathological characteristics were unclear.

### Consensus clustering analysis

The HCC patients were clustered into different subgroups by consensus expression of m^6^A methylation-related genes by using the “Consensus Cluster Plus” R package. The accuracy of clustering results was verified by principal component analysis (PCA). The Kaplan-Meier method calculated OS difference between different clusters. The differences in age, gender, grade and stage among different clusters were assessed using the Wilcoxon rank-sum test or the Kruskal-Wallis test.

### Construction and validation of prognostic model

To identify the prognostic m^6^A methylation-related genes, we used the univariate Cox regression analysis. We then use the machine learning algorithm, LASSO regression analysis,to further eliminate the over-fitting. The m^6^A methylation-related genes that can be used as independent prognostic factors of OS were screened by multivariate Cox regression analysis. All HCC patients were separated into high-risk and low-risk groups based on the median risk score. The survival curve provided a means to compare OS of the two risk subgroups. In addition, the prediction accuracy of the prognostic model was evaluated by a receiver operating characteristic (ROC) analysis.

### Statistical analysis

All statistical analyses were conducted by Perl language and R software 3.6.1. Univariate and multivariate Cox regression analyses, LASSO regression analysis and ROC curve analysis were conducted with R software and the corresponding R packages. For and all comparisons, statistical significance different was defined as a *p *< 0.05.

## Results

### Clinical data of HCC patients

A total of 374 patients with HCC and 50 persons with normal health were included in the TCGA cohort.

### Differentially expressed m6A methylation-related genes

**Figure 1:**
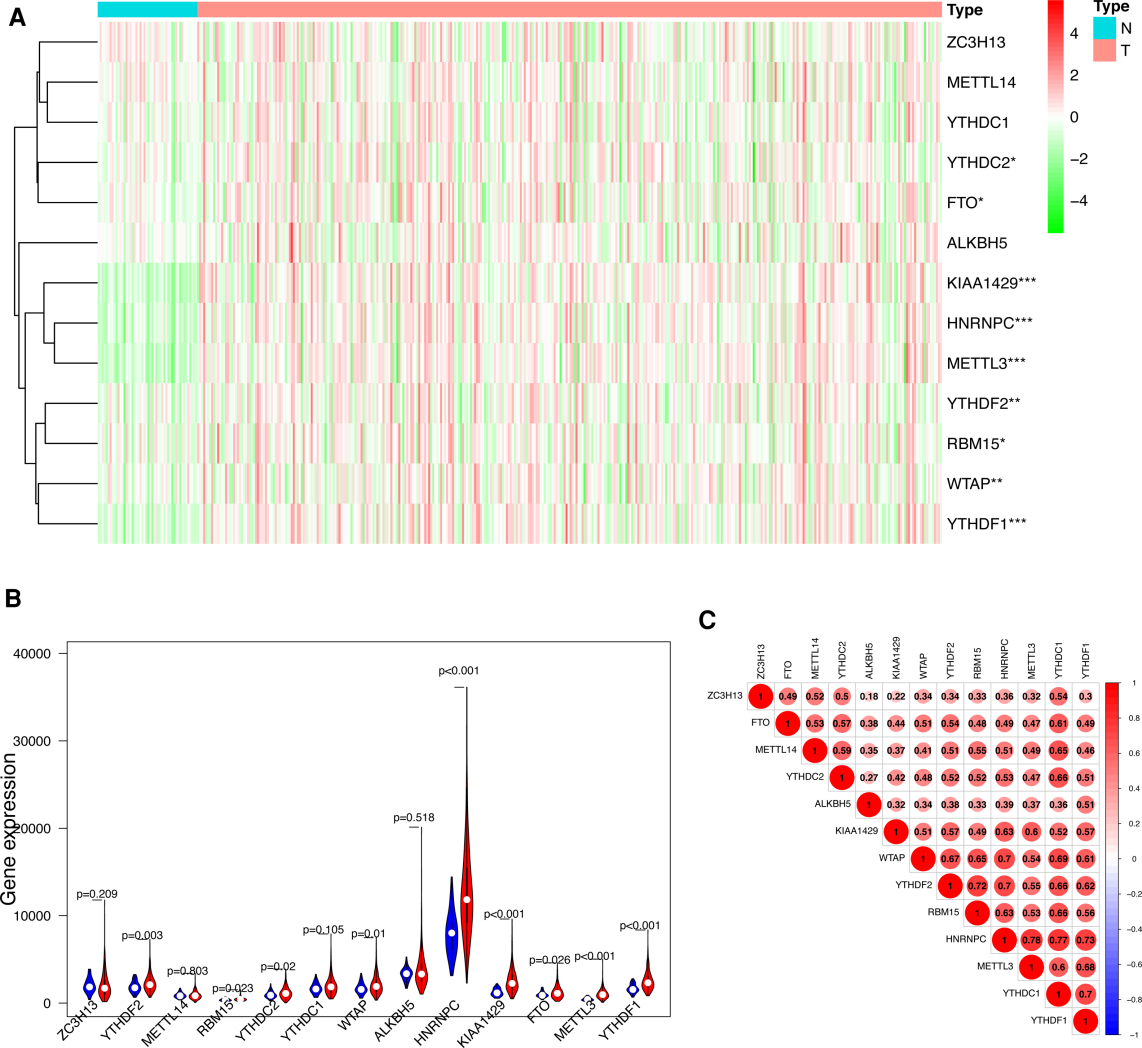
Differentially expressed m6A methylation-related genes between HCC and normal tissues. (**A**) Heatmap of differentially expressed m6A methylation-related genes. (**B**) The expression patterns of differentially expressed m6A methylation-related genes in HCC and normal tissues. Red, tumor tissues; Blue, normal tissues. (**C**) The correlation among m6A methylation-related genes.

We visualized the differential expression of m6A methylation-related genes between tumor tissue and normal tissue with the aid of a heatmap. The expressions of ZC3H13, METTL14, YTHDC1, YTHDC2, FTO, ALKBH5, KIAA1429, RBM15, METTL3, WTAP, YTHDF1 and YTHDF2 were significantly higher in tumor samples than in normal tissue (Figure 1A). In addition, we visualized the expression pattern of the differentially expressed m6A methylation-related genes by volcano plots and box plots (Figure 1B). Pearson correlation analysis showed that YTHDC2 was correlated primarily with HNRNPC (*r* = 0.77) among all interactions of m6A methylation-related genes (Figure 1C).

### Consensus clustering of m6A methylation-related genes

**Figure 2:**
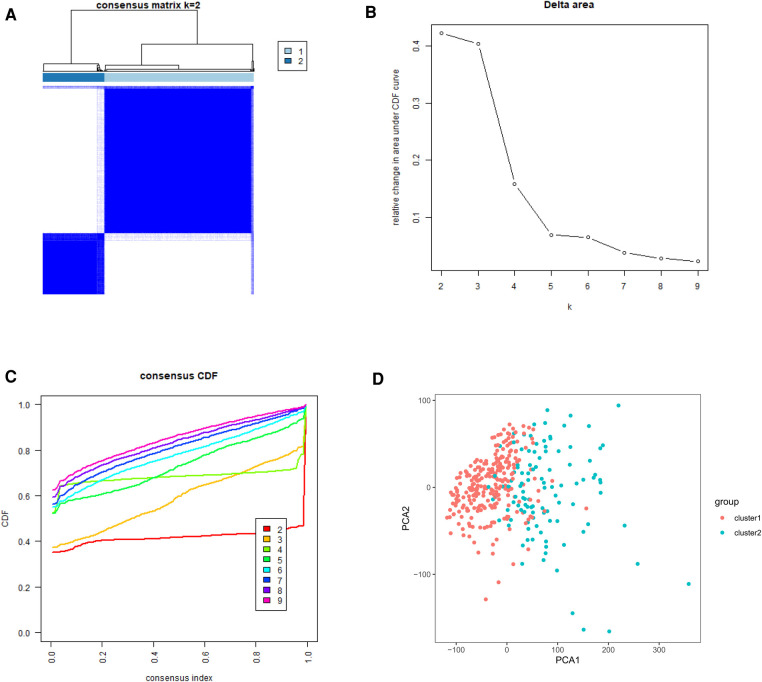
Tumor classification and verification based on m6A methylation-related genes. (**A**) The HCC patients divided into two distinct clusters, *k* = 2. (**B**) Consensus clustering cumulative distribution function for *k* = 2–9. (**C**) Relative change in area under cumulative distribution function curve for *k* = 2–9. (**D**) the principle components analysis based on the m6A methylation-related genes.

**Figure 3:**
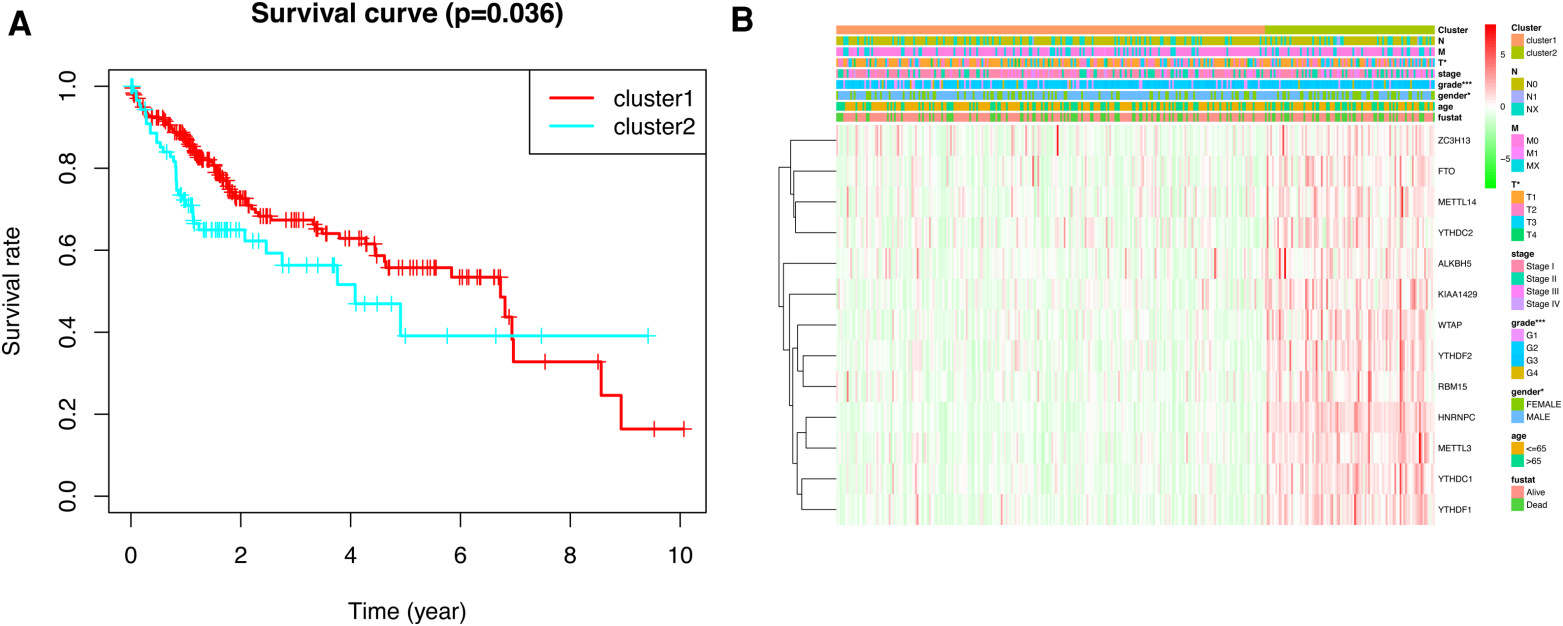
Kaplan–Meier survival analysis and heatmap of expression of m6A methylation-related genes. (**A**) Kaplan–Meier survival analysis of overall survival in different subgroups. (**B**) Heatmap of expression of m6A methylation-related genes and clinicopathological features between different subgroups.

To gain insight into the molecular heterogeneity of HCC and to determine if m6A methylation-related genes presented discernable patterns in HCC, we conducted an unsupervised consensus analysis of all samples. The result of *k* = 2 seemed to be more accurate. It could separate the samples into two subgroups with a lower correlation between them (Figure 2A–C). Then, we used PCA to show the effect of the stratification on the transcriptional profiles of clusters 1 and 2 (Figure 2D). The 5-year OS of cluster 1 was significantly longer than that of cluster 2 (*p *< 0.001) (Figure 3A). Then, the associations between the clustering and clinicopathological features were evaluated. Significant differences in features were found between cluster 1 and cluster 2. These, included grade (*p *< 0.05), gender (*p *< 0.005) and stage (*p *< 0.05) (Figure 3B).

### Construction and validation of prognostic model

**Figure 4:**
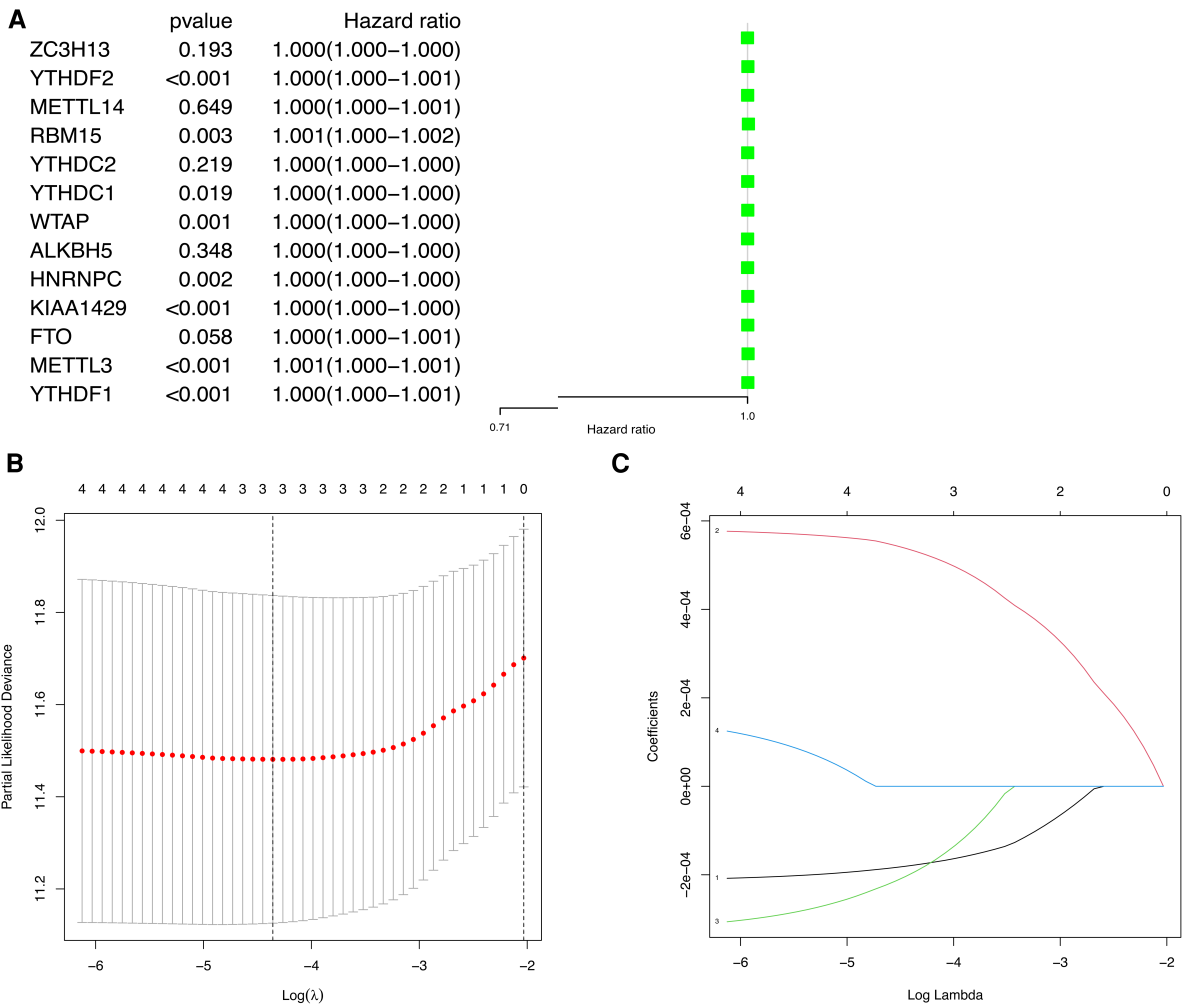
The univariate Cox regression analysis and LASSO regression analysis applied to screening m6A methylation-related genes that optimal used for the construction of the prognostic model. (**A**) The significantly related to survival m6A methylation-related gene by univariate Cox regression analysis. (**B**) Screening of optimal parameter (lambda) at which the vertical lines were drawn. (**C**) LASSO coefficient profiles of the five m6A methylation-related genes with non-zero coefficients determined by the optimal lambda.

To explore the prognostic value of m6A methylation-related genes in HCC, we first conducted a univariate Cox regression analysis to identify genes that are associated with OS (Figure 4A). Then, we undertook a LASSO regression analysis and a multivariate Cox regression analysis to establish an optimal multigene prognostic model for OS.The final result of this was YTHDF2, METTL14, and ZC3H13 (Figures 4B,C). Finally, we use $\mathrm{Risk}\;\mathrm{Score}=\sum_{i=1}^{n}\mathrm{Coe}f_{i}\times X_{i}$ to construct the risk scoring formula (19). Risk score = (YTHDF2 expression × 0.0005313) + (METTL14 expression × −0.0001924) + (ZC3H13 expression × −0.0001777).

**Figure 5:**
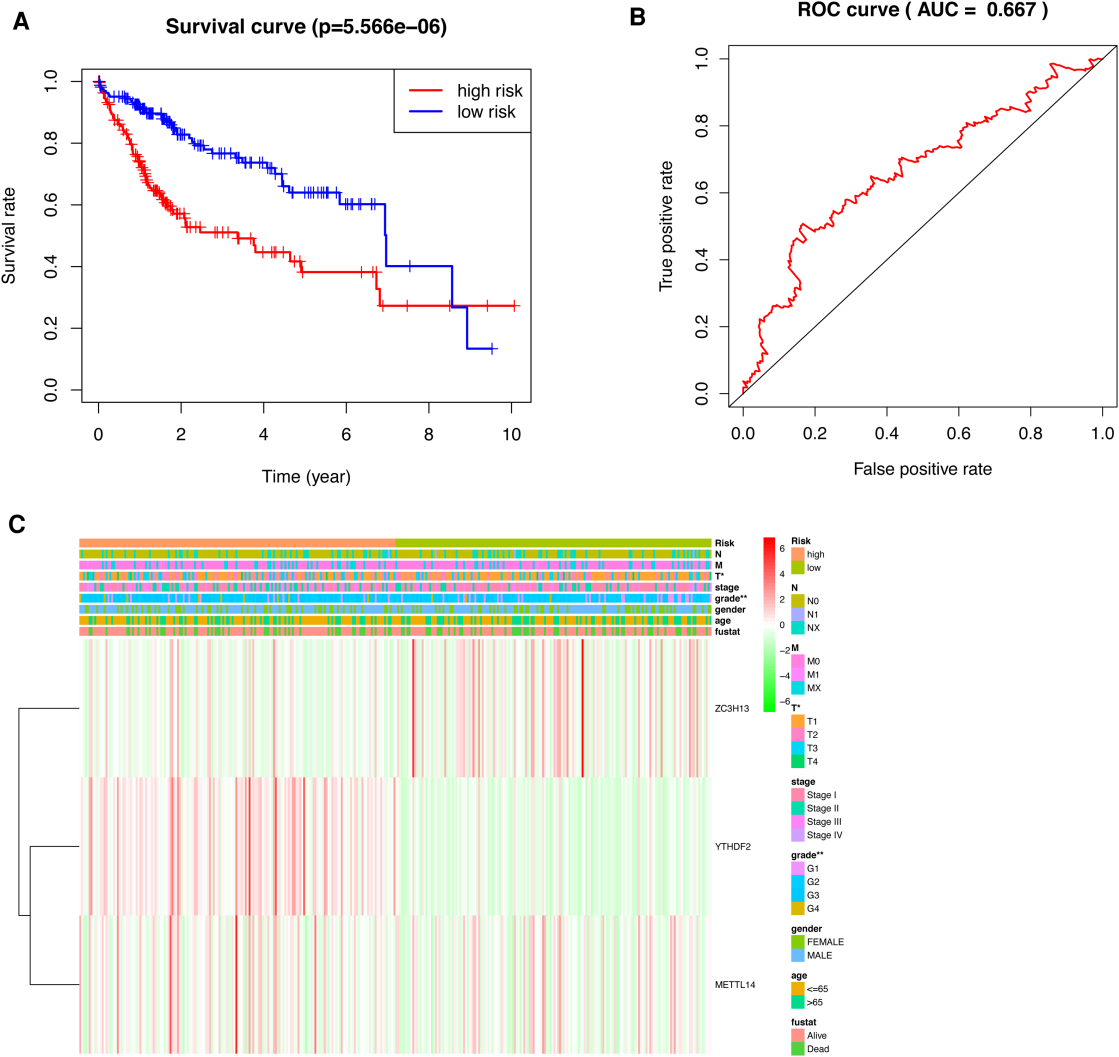
Prognostic model of HCC patients. (**A**) The survival curves for the low-risk and high-risk groups (**B**) ROC curve of OS-related prognostic model in TCGA cohort. (**C**) A risk-clinical correlation heatmap.

HCC patients were separated into low-risk and high-risk groups based on the median risk score. The low-risk group had a higher survival rate than did the high-risk group (*p *< 0.001) according to the survival curves (Figure 5A).In addition, we determined the accuracy of the OS-related prognostic model by constructing a ROC curve (AUC = 0.667) (Figure 5B).

We constructed a risk-clinical correlation heatmap, and found that the grade was correlated to the clinic. At the same time, YTHDF2 and METTL14 were highly expressed in the high risk group, and ZC3H13 was highly expressed in the low risk group (Figure 5C).

**Figure 6:**
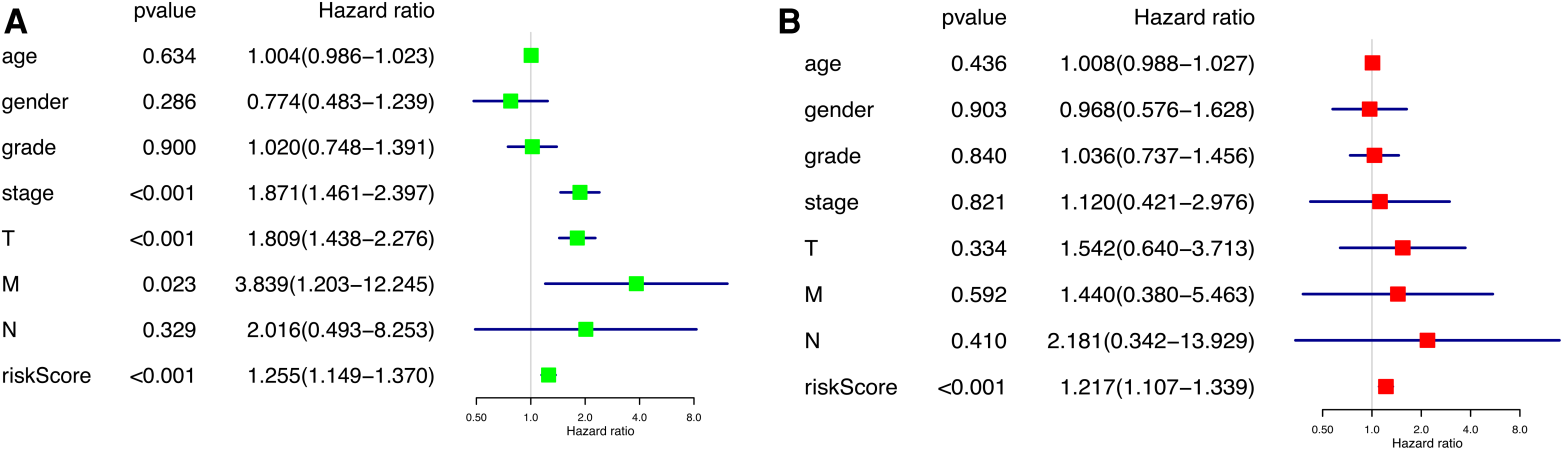
Independent prognostic analysis of prognostic model. (**A**) Univariate factor independent prognostic analysis. (**B**) Multivariate factor independent prognostic analysis.

According to the results of the univariate and multivariate Cox regression analysis (*p *< 0.001), our model can be used as a prognostic factor for HCC (Figures 6A,B).

### Clinical sample validation

**Figure 7:**
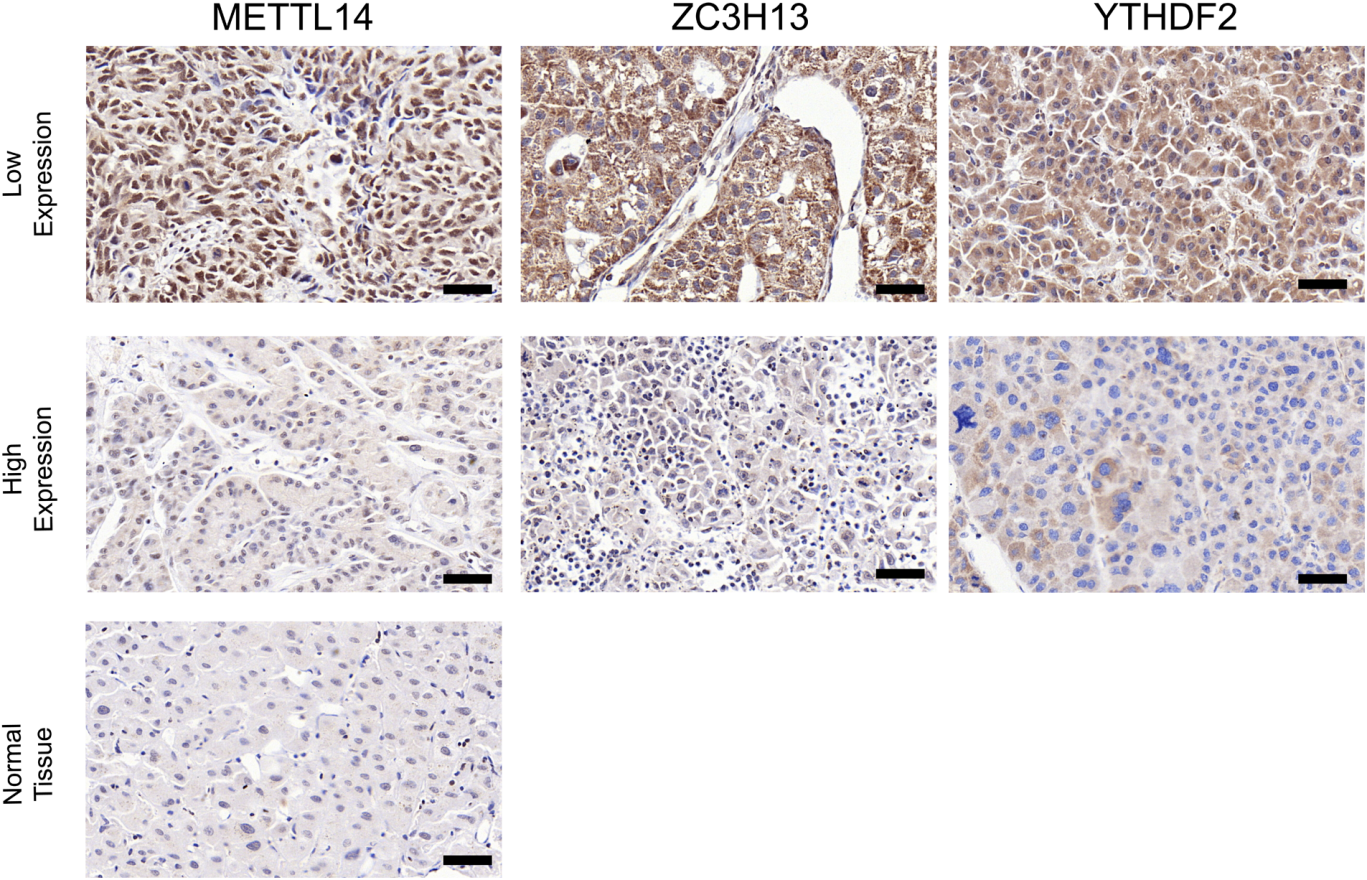
Contrast of paracancerous tissue and tumor tissue immunohistochemical staining. Contrast of paracancerous tissue and tumor tissue immunohistochemical staining (×200) (**A–C**). Intensity comparison of METTL14, YTHDF2, ZC3H13 staining in cancer tissue (**A,B**). Scale bar = 50 µm.

**Figure 8:**
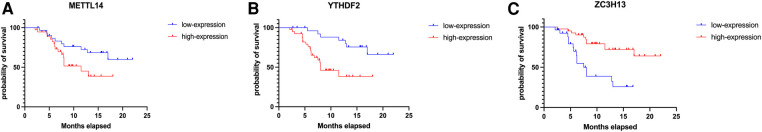
Kaplan–Meier-plotter results of METTL14, YTHDF2, ZC3H13 (**A–C**).

In order to verify what was concluded from the above analysis, we collected pathological specimens from 68 liver cancer surgery patients from the Department of Pathology, Affiliated Hospital of Qingdao University (These specimens had been analyzed and diagnosed as HCC by the Department of Pathology.) We analyzed the specimens for Immunohistochemical staining experiments. Among the 68 HCC specimens that underwent immunohistochemical staining, the cancer tissues were positive for METTL14, YTHDF2 and ZC3H13 staining in comparison to the adjacent tissues, as shown in Figures 7A–C. Among them, 37 specimens (about 54%) were strongly stained in METTL14-related immunohistochemical staining, and 31 (about 46%) were weakly stained. In YTHDF2-related immunohistochemical staining, 40 specimens (about 59%) were strongly stained, and 28(about 41%) were weakly stained. Among ZC3H13-related immunohistochemical staining, 41 cases (about 60%) received strong staining and 27 (about 40%) received weak staining. By collecting the patients' clinical data, we were able to conduct Kaplan-Meier survival analysis. The results showed that patients with low METTL14 expression had survived for a longer time than patients with high METTL14 expression. Also, patients with low YTHDF2 expression had a longer survival time than those with high YTHDF2 expression. Finally, patients with high ZC3H13 expression lived longer than those with low ZC3H13 expression. This result is consistent with the conclusion of bioinformatics analysis, as Figures 8A–C show.

## Discussion

The carcinogenesis of HCC involves an intricate regulatory network. In contrast to the use of a single clinicopathological parameter or gene. It combines diverse biomarkers and establishes that a prognostic model is a more effective way to predict tumor prognosis (20). Currently, m6A methylation has a significant role in various types of cancer. It is ubiquitous in the occurrence and development of cancer (21). A prognostic model that is based on the selected m6A methylation-related genes may be more accurate and effective than a single clinicopathological parameter (21, 22).

In this work, we sought to analyze the relationship between m6A methylation-related genes and the prognosis of HCC patients. We obtained the expression patterns of m6A methylation-related genes in samples from the TCGA database. We identified three m6A methylation-related genes (YTHDF2, METTL14 and ZC3H13) by multivariate Cox regression analysis and used them to construct the prognostic model. Considering our limited data, we will proceed to verify whether this formula is suitable for different groups of people.

YTH N6-methyladenosine RNA binding protein 2 (YTHDF2), the first functionally verified m6A reader, promotes the degradation of m6A-modified mRNAs in humans (23). One study showed that YTHDF2 destabilizes m6A-containing RNA by direct recruitment of the CCR4-NOT deadenylase complex. The latter is recruited to m6A-containing RNAs by direct interaction with the N-terminal region of YTHDF2 (12, 24). YTHDF2 has a great impact on mRNA degradation in different tissues and cell types in vertebrates. In addition, YTHDF2 may act as a tumor suppressor to repress cell proliferation and growth by destabilizing EGFR mRNA in HCC cells. Our results also revealed that YTHDF2 might play an important role in liver cancer development and could be used as a therapeutic target for HCC patients. Considering that the experimental results show that patients with low expression of YTHDF2 live longer than patients with high expression of YTHDF2, we will continue to follow related reports and studies. Methyltransferase like 14 (METTL14), an RNA methyltransferase, has been implicated in mRNA decay, biogenesis, and translation control through m6A modification (25). Some studies have shown that METTL14-mediated m6A modification is involved in regulating numerous genes in various types of cancer (26–29). Moreover, one study reported that crosstalk between METTL14 and miR-186 regulates hepatoblastoma progression by means of the Wnt/β-catenin signaling pathway (30). Another study showed that METTL14 promotes the progression of HCC by m6A-mediated upregulation of microRNA-873-5p (31). Our observations suggest that METTL14 abnormalities contribute to an increased risk of developing HCC. Zinc finger CCCH-type containing 13 (ZC3H13), a canonical CCCH zinc finger protein, plays an important role in modulating RNA m6A methylation in the nucleus. One report stated that ZC3H13 suppresses colorectal cancer proliferation and invasion by inactivating Ras-ERK signaling. However, there are few studies of the mechanism of ZC3H13 in cancer. Our findings suggest that ZC3H13 is a promising diagnostic marker in HCC.

In addition, the expression of m6A methylation-related genes was examined in samples from other databases. YTHDF2, METTL14, and ZC3H13 were highly expressed in HCC tissues and closely related to OS in HCC patients. Based on existing reports, the mechanism of YTHDF2, METTL14, and ZC3H13 in HCC is still not clear. It requires further research and exploration. Our study used TCGA database to establish a prognostic model related to YTHDF2, METTL14, and ZC3H13 expression for predicting the prognosis of HCC patients. This would undoubtedly provide a new treatment strategy for the treatment of HCC patients. However, our research still has certain limitations. First, this is a retrospective study. Thus, there may be biases in to the respect to the choice of variables. Second, the mechanisms of the action of the m6A methylation-related genes in HCC need to be validated in *in vivo* and *in vitro* experiments in order to confirm our results.

In conclusion, we have constructed a multigene model for OS that can predict the prognosis of HCC patients and provide new treatment strategies for HCC.

The study was approved by the the Affiliated Hospital of Qingdao University,written informed consent was obtained. The study was performed in accordance with the Declaration of Helsinki.
